# Shenhua Tablet inhibits mesangial cell proliferation in rats with chronic anti-Thy-1 nephritis

**DOI:** 10.1186/s40659-016-0078-3

**Published:** 2016-03-11

**Authors:** Wenjia Geng, Ribao Wei, Shuwen Liu, Li Tang, Hanyu Zhu, Pu Chen, Jie Wu, Xueguang Zhang, Fei Zhu, Zhong Yin, Xiangmei Chen

**Affiliations:** Department of Nephrology, Chinese PLA General Hospital, Chinese PLA Institute of Nephrology, State Key Laboratory of Kidney Diseases, National Clinical Research Center for Kidney Diseases, Beijing Key Laboratory of Kidney Disease Research, Beijing, 100853 People’s Republic of China; Department of Nephrology, Guangdong Provincial Hospital of Chinese Medicine, Guangzhou, 510120 People’s Republic of China

**Keywords:** Shenhua Tablet, Chronic anti-Thy-1 glomerulonephritis, Mesangial cell proliferation, Erk

## Abstract

**Background:**

In China, mesangial proliferative glomerulonephritis (MsPGN) is one of the most common kidney diseases. In this study, we treated a rat model of chronic anti-Thy-1 MsPGN with Shenhua Tablet and evaluated whether the tablet was able to protect the kidney function. Thirty-six Wistar rats were randomly divided into six groups: (1) Sham surgery (Sham); (2) anti-Thy-1 nephritis model (Thy-1); (3) anti-Thy-1 nephritis model + irbesartan-treated (Irb); (4) anti-Thy-1 nephritis model + low-dose of Shenhua Tablet (SHL); (5) anti-Thy-1 nephritis model + medium-dose of Shenhua Tablet (SHM); (6) anti-Thy-1 nephritis model + high-dose of Shenhua Tablet (SHH).

**Results:**

Thirteen weeks after drug treatment, urinary proteins were quantified and renal pathological changes were thoroughly examined at the time point of 24 h. Meanwhile, the expression levels of p-Erk1/2, cyclin D1 and p21 at the renal cortex were also tested. The levels of urinary proteins and total cholesterol in the blood were significantly reduced in rats treated with any drug tested in this study. The level of triglyceride was significantly reduced in all three Shenhua Tablet-treated groups. Renal pathomorphological scores were significantly improved in groups of Irb, SHM and SHH. Mesangial cell proliferation was significantly inhibited in any drug-treated group. p-Erk1/2 and cyclin D1 were downregulated whereas p21 was upregulated in the renal cortex.

**Conclusions:**

Our study indicated that Shenhua Tablet is able to inhibit the abnormal proliferation of mesangial cells and to prevent kidney damage, which is likely associated with downregulation of p-Erk1/2 and reduced activity of its downstream target-cyclin D1.

## Background

Mesangial proliferative glomerulonephritis (MsPGN) is characterized by widespread mesangial cell proliferation and an accumulation of extracellular matrix, and is the most common type of glomerulonephritis globally [1, 2]. Abnormal proliferation of mesangial cells plays an important role in the onset and progression of glomerlular lesion [3]. Abnormally proliferative mesangial cells are able to release mediators of inflammation and mesangial matrix, which often cause severe glomerulosclerosis and interstitial fibrosis, resulting in irreversible progressive glomerulosclerosis, eventually leading to end-stage renal disease (ESRD) [4]. Therefore, to inhibit mesangial cell proliferation is a great therapeutic strategy for MsPGN and searching for such drugs is urgently needed for the treatment of this disease. Clinically, MsPGN patients especially IgAN patients, are currently treated with drugs such as hormones, cyclophosphamide, azathioprine, mycophenolate mofetil, leflunomide, and so on. However, these drugs often have toxic side effects, which largely compromise their clinical applications. Therefore, it has become critical to seek for effective treatments actively and further understand the mechanism underlying how these drugs protect the kidney functions [5, 6].

Shenhua Tablet has been widely used in clinical settings for many years. Clinical evidence has shown that this drug is able to reduce the level of proteinuria in patients with IgA nephropathy, and greatly improve the life quality of patients without serious adverse reactions [7]. However, whether Shenhua Tablet can protect the kidney functions of MsPGN patients remains completely unclear. This study aimed to investigate the role of Shenhua Tablet in the treatment of chronic anti-Thy-1 MsPGN, and to explore the possible mechanism underlying the improvement in kidney function after Shenhua Tablet treatment.

## Results

### Shenhua Tablet reduces the proteinuria level in rats with chronic anti-Thy-1 nephritis

**Fig. 1:**
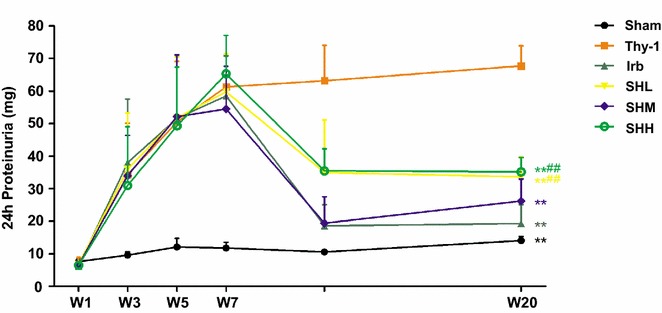
The levels of urinary protein in 24 h were monitored for each group over the experimental process (week 1, 3, 5, 7, 11 and 20 after injection of Thy-1 antibody). At the last time point W20, the urinary proteins in 24 h in all drug-treated groups were significantly lower (*p* < 0.01) than that in the Thy-1 group. Values were expressed as mean ± SD. * *p*< 0.05, ** *p*< 0.01 vs. Thy-1; ^#^ *p*< 0.05, ^##^ *p*< 0.01 vs. Irb group

The levels of urinary protein in 24 h were recorded for each group over the experimental process. We observed that the urinary proteins were gradually increased in each group after being given Thy-1 antibody four times through intravenous injection at the time points W1, W3, W5 and W7 (*p* < 0.05). At the time point W11 (21 days after the last injection of Thy-1 antibody at W7), urinary proteins in 24 h in experimental groups were still significantly higher than that in the Sham group, indicating that the animal model of chronic anti-Thy-1 nephritis was reliably established [8, 9]. At the last time point W20, the urinary proteins in 24 h in all drug-treated groups were significantly lower (*p* < 0.01) than that in the group of anti-Thy-1. However, there was no significant difference between SHM group and Irb group in terms of levels of urinary proteins in 24 h (*p* = 0.14), which suggested that SHM and Irb functioned similarly in reducing the urinary proteins in 24 h for experimental rats (Fig. 1).

### The effect of Shenhua Tablet on the renal function and biochemical indices in blood

**Fig. 2:**
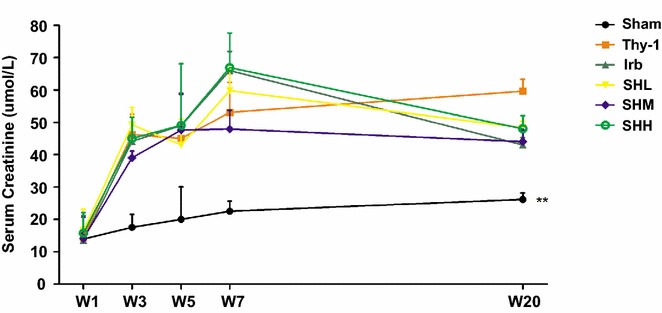
The levels of serum creatinine in each groups at different time points. The levels of serum albumin were quantified in all six groups at different time points (week 1, 3, 5, 7 and 20 after injection of Thy-1 antibody). Values were expressed as mean ± SD. * *p*< 0.05, ** *p*< 0.01 vs. Thy-1

The levels of serum creatinine in each group were also determined at the last time point W20. As observed, the level of serum creatinine in anti-Thy-1 group was significantly higher compared with that in the Sham group (*p* = 0.003). However, the levels of serum creatinine were not significantly changed (*p* > 0.05) in all drug-treated groups in contrast to that in the Thy-1 group, which demonstrated that irbesartan and Shenhua Tablet of any dose in this study were unable to reduce the level of serum creatinine in rats with anti-Thy-1 nephritis (Fig. 2).

**Fig. 3:**
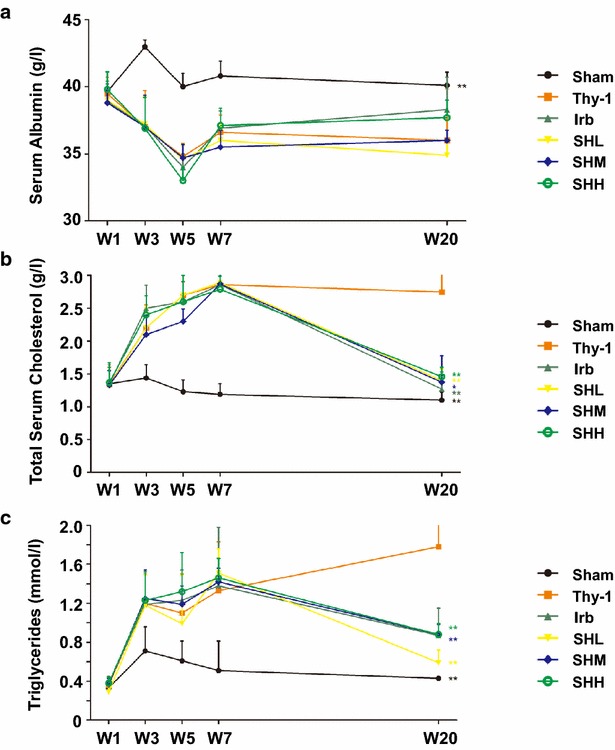
The effect of Shenhua Tablet on the biochemical indices in blood. **a** The levels of serum albumin were quantified in all six groups at different time points (week 1, 3, 5, 7 and 20 after injection of Thy-1 antibody). **b** The levels of total cholesterol in blood were quantified in all six groups at different time points mentioned above. **c** The levels of triglyceride were quantified in all six groups at different time points mentioned above. Values were expressed as mean ± SD. * *p*< 0.05, ** *p*< 0.01 vs. Thy-1. n = 6

At W20, the levels of serum albumin were not significantly increased (*p* > 0.05) whereas the levels of total cholesterol in blood were strikingly reduced (*p* < 0.05) in all drug-treated groups, compared with those in the Thy-1 group. However, the levels of total cholesterol were not significantly different among all drug-treated groups (*p* > 0.05). It was noteworthy that Shenhua Tablet of any dose (low, medium or high) was able to reduce the levels of triglyceride significantly (*p* < 0.05). However, there was no significant differences when treated with different doses of the drug (*p* > 0.05) (Fig. 3).

### Shenhua Tablet inhibits proliferation of mesangial cells in rats with anti-Thy-1 nephritis and improves renal pathological scores

**Fig. 4:**
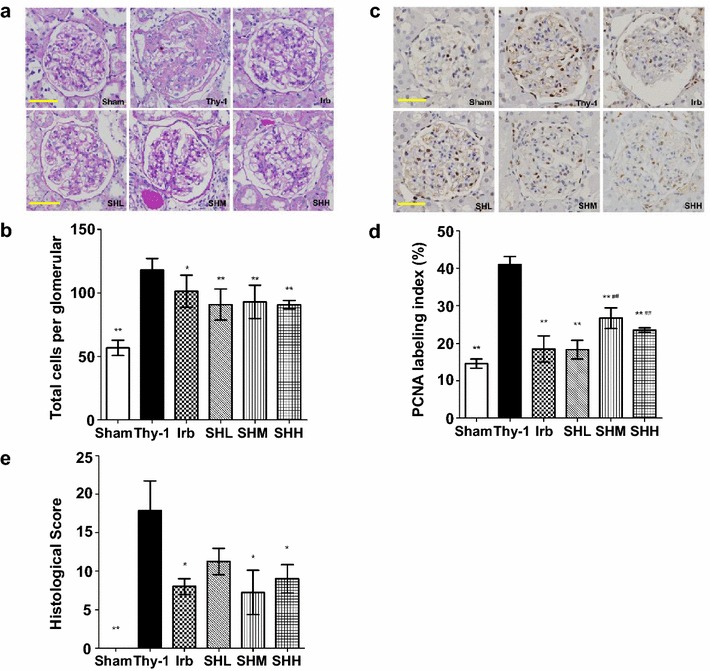
Shenhua Tablet inhibits proliferation of mesangial cells in rats with anti-Thy-1 nephritis and improves renal pathological scores. **a** Histological changes in PAS-stained sections at W20 (×400 magnification). *Scale bar* 50 µm. **b** The total number of glomerular cross section in the six groups. **c** PCNA-positive cells in the groups at W20. *Scale bar* 50 µm. **d** The PCNA labeling index in all six groups. The index was calculated according to the ratio of PCNA positive cells to total glomerular cells. **e** Histological scores of the renal lesions in all six groups. Values were expressed as mean ± SD. * *p*< 0.05, ** *p*< 0.01 vs. Thy-1

The histopathological changes in glomeruli were examined under the microscope after PAS staining. At W20, the changes including increased proliferation of glomerular cells (*p* < 0.01) and mesangial cells, accumulation of mesangial matrix, widening of mesangial region, compression, deformation even occlusion of the capillary lumen, were observed in the Thy-1 group but not in the Sham group (Fig. 4a, b). Thirteen weeks after drug treatment, the proliferation of mesangial cells was significantly reduced; the widening of mesangial regions and accumulation of mesangial matrix were strikingly improved and the capillary lumen also recovered back to normal as compared with those in the Thy-1 group (Fig. 4a). No increase in the number of mesangial cells and accumulation of mesangial matrix were observed in the Sham group (Fig. 4a). At W20, the number of glomerular cells in all drug-treated groups, including Irb (*p* < 0.05), SHL (*p* < 0.01), SHM (*p* < 0.01) and SHH (*p* < 0.01), was significantly reduced compared with that in the anti-Thy-1 group (Fig. 4a, b).

PCNA (proliferating cell nuclear antigen) is well-known as a marker of cell proliferation which localizes in the nucleus. PCNA is barely detectable in the glomeruli of the Sham group. However, it was significantly increased at W20 in the Thy-1 group, compared with the Sham group (*p* < 0.01). Thirteen weeks after drug treatment, PCNA-positive cells were significantly reduced in all drug-treated groups when compared with those in the Thy-1 group (*p* < 0.01) (Fig. 4c, d).

Renal pathological score is generally used to comprehensively evaluate the kidney damage. At W20, the score was significantly higher in the Thy-1 group compared with that in the Sham group (*p* = 0.001); However, this score was significantly decreased in three drug-treated groups, including Irb (*p* = 0.01), SHM (*p* = 0.014) and SHH (*p* = 0.017), when compared with that in the anti-Thy-1 group (Fig. 4e).

### Shenhua Tablet downregulates p-Erk1/2 and cyclin D1 while upregulating p21 in the renal cortex of rats with anti-Thy-1 nephritis

**Fig. 5:**
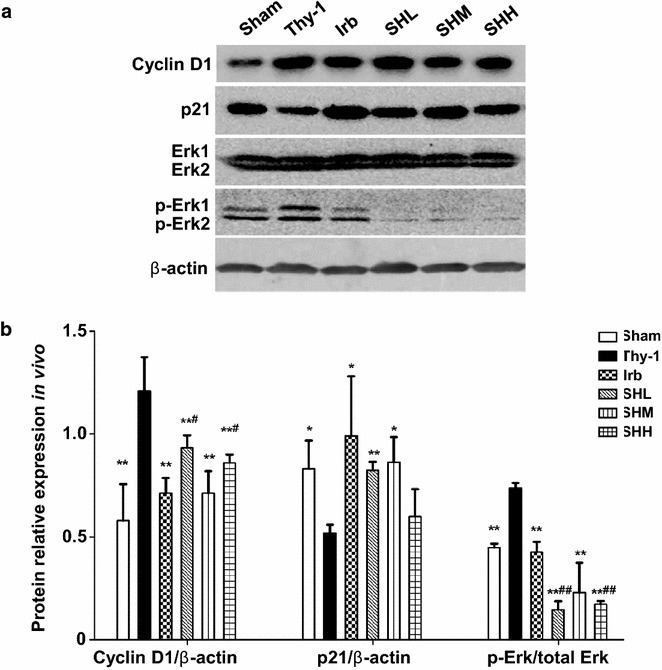
Shenhua Tablet downregulates p-Erk1/2 and cyclin D1 while upregulating p21 in the renal cortex of rats with anti-Thy-1 nephritis. **a** Western blot was performed to detect the expression levels of p-Erk1/2, cyclin D1 and p21 in all drug-treated groups as well as the controls. **b** Quantification of the cyclin D1, p21 and p-Erk1/2. Expression level of p-Erk1/2 was normalized with total Erk1/2 while cyclin D1 and p21 were normalized with beta-actin. Each *bar* represents the mean ± SD of six rats. * *p*< 0.05, ** *p*< 0.01 vs. Thy-1; ^#^ *p*< 0.05, ^##^ *p*< 0.01 vs. Irb group

As Shenhua Tablet was able to inhibit the proliferation of mesangial cells, improve pathological scores and reduce the proteinuria level in rats with anti-Thy-1 nephritis, we then went on to explore the possible molecular mechanism underlying how this drug worked. We first investigated the expression level of p-Erk1/2 to see whether Erk/Mapk signaling pathway was activated; we also examined cyclin D1 and p21 to see whether these cell cycle regulatory proteins were dysregulated. The data indicated that expression levels of p-Erk and cyclin D1 were strikingly increased in the Thy-1 group (*p* < 0.01) while the level of p21 was largely reduced (*p* < 0.05) when compared with the Sham group. However, in all groups with drug treatment, the expression levels of p-Erk were significantly reduced compared to that in the anti-Thy-1 group (*p* < 0.01). It is noteworthy that the expression levels of p-Erk in all Shenhua Tablet-treated groups were even more reduced compared with that in the Irb-treated group. We also observed that in the drug-treated groups, the expression level of cyclin D1 was significantly decreased, compared with that in the Thy-1 group (*p* < 0.01). Moreover, we noticed that the expression of cyclin D1 was similar between the Irb group and SHM group, which suggested that Irb and Shenhua Tablet with medium dose had the same effect on the expression of cyclin D1. In addition, the expression levels of p21 in groups of Irb, SHL and SHM were significantly higher than that in Thy-1 group (*p* < 0.05) while there was no significant difference among all drug-treated groups (*p* > 0.05) (Fig. 5).

## Discussion

Abnormal proliferation of mesangial cells is an important pathological feature of multiple immune-mediated glomerular diseases; continuous mesangial cell proliferation eventually leads to irreversible glomerulosclerosis, and even progresses to renal failure [4]. MsPGN, including IgAN and non-IgA MsPGN, is the most common glomerulonephritis and the primary cause of end-stage renal disease (ESRD) [10]. Therefore, inhibition of mesangial cell proliferation is a great strategy to treat proliferative glomerular diseases. Hence, searching for such inhibitors can have important clinical implications.

In the past 20 years, although mesangial cell proliferation signaling pathways have become clearer, specific drugs used to inhibit the proliferation of mesangial cells are still poorly available. Currently, clinical applications are only limited to a few drugs, such as hormones, cytotoxics, and so on. With the development of Chinese medicine theory, a number of classical prescriptions, special prescriptions and drugs, patent medicines, and their extracts or injections have been proved to be effective in the treatment of human diseases in some respects, which indicates traditional Chinese medicine can also be used in the treatment of chronic kidney disease, such as MsPGN. With accumulating evidences and more studies in clinical and basic research, the efficacy of Chinese medicine in the treatment of chronic kidney diseases has been well recognized in the national and international medical field. It has been clear that Chinese medicine and integrative medicine are effective approaches to treat patients with chronic kidney disease (including MsPGN). Data from multi-center, randomized, double-blind and placebo-controlled clinical experiments have shown that Shenhua Tablet is able to reduce proteinuria level in IgA nephropathy patients deficient with Qi and Yin, and to improve their clinical symptoms without serious adverse reactions [7]. However, the mechanism underlying remains poorly understood. To date, only a few studies show that through toll-like receptors (TLRs), treatment of Shenhua Tablet is able to reduce the secretion of inflammatory cytokines, thereby improving the ischemia-reperfusion-induced acute kidney injury [11]. Currently, no experimental data demonstrate whether Shenhua Tablet is able to inhibit mesangial cell proliferation in rats with anti-Thy1 nephritis.

In this study, we first report that Shenhua Tablet is able to inhibit mesangial cell proliferation and protect kidneys in rats with chronic anti-Thy-1 MsPGN, a classic animal model widely used for human MsPGN. The greatest feature of this model is that the renal damages are irreversible, which is different from the acute anti-Thy-1 MsPGN model in which mesangial cell proliferation and matrix accumulation are reversible. To some extent this model can mimic human chronic MsPGN [12], which is an important consideration to improve ‘bench to bedside’ applicability. However, chronic anti-Thy-1 nephritis in the rat is caused by abnormal deposition of IgG in glomeruli while human IgAN, the most common form of MsPGN, is primarily caused by the deposition of IgA in the glomeruli [13], together with the species and genetic differences between rat and human, therefore the results obtained from this model can not be directly used to predict human IgAN. Regardless of this limitation, so far anti-Thy-1 nephritis model is the most representative animal model used to search for drug targets against mesangial cell proliferation [9].

To perform an in vivo study, we orally treated the rats with Shenhua Tablets (low, medium and high dose) once a day for 13 weeks and assessed the drug effect. The data showed that Shenhua Tablets was able to reduce the level of proteinuria and total cholesterol significantly, which was comparable to the therapeutic effect observed in the positive control-irbesartan group. In this study, we noticed that the level of serum creatinine in the rats with anti-Thy-1 nephritis was not significantly reduced when Shenhua Tablet of any dose was applied. In the future study, we may extend the time for drug treatment following the time point of W20, and re-examine if the level of creatinine becomes significant different between the drug-treated group and anti-Thy-1 group. We are also interested in testing whether protein uptake in the proximal tubules is increased in our future studies. We also found that Shenhua Tablet could strikingly inhibit glomerular cell proliferation based on the data obtained from renal pathological scores, glomerular cell counting, PCNA-positive glomerular cell counting, and so on. Previous studies have shown that ERK activity plays an important role in mesangial cell proliferation, and this activity is also essential to cell cycle progression [14, 15]. ERK can translocate into the nucleus, phosphorylate and activate a number of transcription factors, such as AP-1, ELK-1, SAP, and then promote cell proliferation [16]. Once the ERK/MAPK pathway is activated, the downstream target genes, such as cyclin D1, an important regulator of cell cycle at the G1 phase [17], will be activated as well. Furthermore, another important cyclin, termed CDK4, needs to complex with its regulatory subunit cyclin D1 to be functional [18, 19]. In acute anti-Thy-1 nephritis model, cyclin D1, CDK4 and other cell cycle-related proteins are all upregulated when mesangial cells are abnormally proliferating. In vitro experiments also confirmed that mesangial cell proliferation was strikingly inhibited when the expression level of cyclin D1 was decreased following removal of the serum or PDGF stimulation [19]. Therefore, we examined one of the key proteins in the Erk/Mapk signaling pathway-Erk1/2, and the cell cycle regulatory proteins in the Shenhua Tablet-treated groups. We found that treatment with Shenhua Tablet downregulated the phosphorylation of Erk1/2 and cyclin D1 while upregulating p21 in the rat model. Therefore, the possible mechanism underlying Shenhua Tablet inhibits the mesangial cell proliferation is that this drug specifically deactivates Erk in the Erk/Mapk pathway, thus downregulates cell cycle regulatory proteins, and ultimately inhibits cell proliferation. These findings have offered a therapeutic potential to Shenhua Tablet in the treatment of chronic glomerulonephritis, particularly for those MsPGN-derived diseases, such as IgAN. In addition, patients with chronic kidney disease often have an abnormal metabolism in blood lipids [20]. Lipids have a direct role in glomerular injury, and may also result in glomerular mesangial cell activation and proliferation [21], and dyslipidemia has been known to be one of the risk factors for the deterioration of renal function. Interestingly, we also found that Shenhua Tablet with different doses could all significantly reduce the levels of both triglycerides and total cholesterol whereas the positive control Irb could only reduce the level of total cholesterol but not of triglycerides. The molecular mechanism underlying this observation needs to be further explored.

## Conclusions

In this study, we discovered that Shenhua Tablet is able to reduce the level of 24 h proteinuria and the degree of kidney injury in the chronic anti-Thy-1 nephritis rat model. These functional improvements by Shenhua Tablet treatment in the injured kidneys might be directly associated with the deactivation of Erk signaling pathway, modulations of downstream cell cycle regulatory proteins, thereby inhibiting the abnormal proliferation of mesangial cells.

## Methods

### Pharmaceutical preparation

Compound Shenhua Tablets (0.6 g/tablet, extracted from at least 3.095 g of crude drug) were nicely provided by Hunan Traditional Chinese Medicine Research Institute (Batch No. 20090910). The main components in this tablet include Radix Astragali, Fruit of Glossy Privet, Curcuma and Honeysuckle. The non-standardized product number in People’s Liberation Army is F01012 (2006). irbesartan was provided by Hangzhou Sanofi-Aventis Minsheng Pharmaceutical Co., Ltd. (Batch number: 0A008; approval number: Zhunzi J20080061). The drug powder was mixed and suspended with 0.4 % CMC-Na (Wako Pure Chemical Industries, Ltd., Japan) solution prior to intragastric administration to rats.

### Animal model of chronic anti-Thy-1 nephritis

Seven-week-old male Wistar rats (SPF grade, ~200 g) were purchased from Beijing Vital River Laboratory Animal Technology Co., Ltd., and were bred in the animal room (cleaning grade) of Experimental Animal Center of the People’s Liberation Army General Hospital. All animal experiments were conducted in accordance with the provisions of Experimental Animal Committee of the People’s Liberation Army General Hospital.

**Fig. 6:**
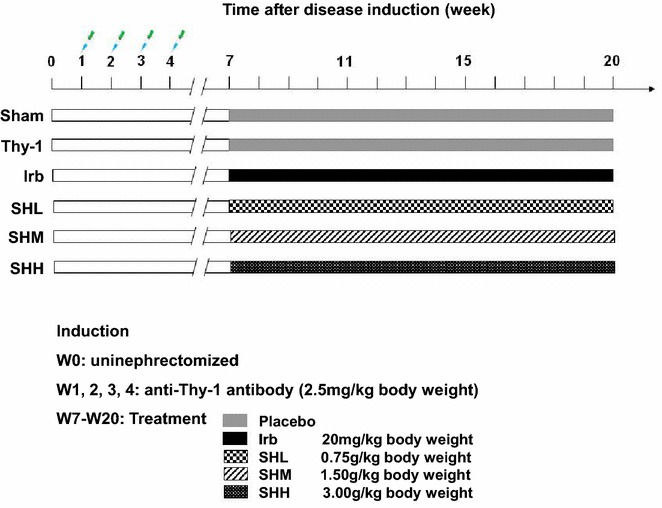
A scheme of drug treatment for the animal model of chronic anti-Thy-1 MsPGN. Thirty-six Wistar rats were randomly divided into six groups: (1) Sham surgery group (Sham); (2) anti-Thy-1 nephritis model group (Thy-1); (3) anti-Thy-1 nephritis model + irbesartan-treated group (Irb); (4) anti-Thy-1 nephritis model + low-dose of Shenhua Tablet group (SHL); (5) anti-Thy-1 nephritis model + medium-dose of Shenhua Tablet group (SHM); (6) anti-Thy-1 nephritis model + high-dose of Shenhua Tablet group (SHH)

Rats were randomly divided into six groups as followed: (1) Sham control (not nephrectomized, injected with same dose of 1 × PBS); (2) Thy-1 (nephrectomized, injected with Thy-1 antibody); (3) Irb (nephrectomized, injected with Irb at the dose of 20 mg/kg body weight); (4) SHL (nephrectomized, injected with Shenhua Tablet at the dose of 0.75 g/kg body weight); (5) SHM (nephrectomized, injected with Shenhua Tablet at the dose of 1.5 g/kg body weight) and (6) SHH (nephrectomized, injected with Shenhua Tablet at the dose of 3 g/kg body weight). Each group has six mice. Seven days after unilateral nephrectomy [22], the mice were given monoclonal Thy-1 antibody (2.5 mg/kg) via intravenous injection [23, 24], which was performed once a week for 4 weeks [25] to induce chronic MsPGN. Twenty-one days after the last injection of Thy-1 antibody (W7), rats were daily treated with drugs (Irb or Shenhua Tablet) at the dose indicated above by gavage for 13 weeks. At each experimental time point, the Sham group was treated with CMC-Na solution alone for 13 weeks as well (Fig. 6).

Biochemical and histopathological approaches were taken to trace the progression of MsPGN for each group after the disease was induced by Thy-1 antibody. Urinary protein and serum creatinine in 24 h were tested every 2 weeks. At the 18th week (the 13th week after drug intervention), the 24 h urine was collected and the rats were then anesthetized with 2 % sodium pentobarbital (45 mg/kg) by intraperitoneal injection. The rats were dissected and 8 ml of blood was collected to assess renal function and biochemical indicators of blood. The kidney was cut into several pieces; One of them was fixed with 4 % paraformaldehyde/PBS for pathological examination and another piece saved for immunostaining was embedded in OCT (Optimal Cutting Temperature compound) and promptly frozen in liquid nitrogen and then stored at −80 °C; The rest of the kidney was directly frozen in liquid nitrogen and then stored at −80 °C. The changes in proteinuria, renal function and pathology were examined and the expression levels of p-Erk1/2, cyclin D1 and p21 in the renal cortex were also tested.

### Renal pathological examination

Kidneys were first fixed with 4 % paraformaldehyde/PBS buffer, dehydrated with ethanol and then embedded in paraffin. The kidneys were sectioned into slices of 4 μm thickness and PAS (Periodic Acid-Schiff) staining was performed to examine pathological changes under light microscope. The degree of renal impairment was semi-quantitatively and double-blindly scored by two pathologists following the published scoring system [26–29]. We randomly selected ten separate field for each specimen to evaluate lesions (400× magnification), and averaged the values for the histological score.

### Immunohistochemical analysis

The paraffin sections were fixed on polylysine-coated slides, deparaffined in xylene, and then rehydrated in ethanol of decreasing concentrations, and finally rinsed in water. Endogenous peroxidase was blocked with 3 % hydrogen peroxide at room temperature. The slices were then immersed in sodium citrate buffer (pH 6.0) and microwaved for 10 min. After being blocked with 1.5 % normal goat serum and incubated at room temperature for 20 min, the sections were stained with PCNA (Abcam; mouse monoclonal; 1: 500 dilution) antibody and incubated overnight at 4 °C. The following day, the unbound primary antibody was washed away with 1 × PBS, then biotin-labeled secondary antibody was added and incubated at room temperature for 40 min. After being washed with 1 × PBS, horseradish peroxidase conjugated streptavidin (Vectastain Elite ABC kit, Vector Labs, USA) was added and incubated at room temperature for an additional 40 min. After PBS wash, the sections were treated with DAB (chromogenic reagent) for 5 min until the color turned brown and were then restained with hematoxylin as a last step. Kidney sections from three rats at each time point were used to quantify proliferative glomerular cells under high magnification microscope (400×) in a blind manner. Ten glomerluli were examined for each section. PCNA-labeling index was calculated as “the number of PCNA-positive cells divided by the total number of glomerular cells”.

### Western blotting

The kidneys were lysed in RIPA buffer (50 mmol/L Tris-HCl, 150 mmol/L NaCl, 0.5 % deoxycholate, 1 % Nonidet P-40, 0.1 % SDS, 0.5 mM PMSF, 1 μg/ml leupeptin and 1 μg/ml aprotinin) on ice for 30 min, then centrifuged at 12,000 rpm at 4 °C for 30 min. The protein concentration was measured with Pierce BCA assay kit (Thermo Fisher Scientific, USA). Sixty to one-hundred μg of total protein were loaded and run with 12 % polyacrylamide gel. After electrophoresis, proteins were transferred onto nitrate cellulose membrane. The membrane was first blocked with 5 % skim milk at room temperature for 1 h, and then incubated overnight at 4 °C with the primary antibodies against p-Erk1/2 (mouse monoclonal, 1: 1000 dilution; Beyotime Institute of Biotechnology, China), cyclin D1 (mouse monoclonal, 1: 100 dilution; Santa Cruz, USA), p21 (mouse monoclonal, 1: 5000 dilution; Novus Biologicals, USA) or β-actin (mouse monoclonal, 1: 5000 dilution; Sigma, USA) respectively. After incubation of individual primary antibody, the membrane was washed three times with TBST (Tris-Buffered Saline plus Tween 20) followed by the incubation of secondary antibody for 1 h. The membrane was washed with TBST again and was then detected using an enhanced chemiluminescence (ECL) method. The intensity of proteins was quantified with Quantity One software (Bio-Rad, USA). All experiments were repeated at least three times.

### Statistical analysis

All data were processed with SPSS 15.0 software (SPSS Inc. USA). Charts were generated with Excel 2003. Data from continuous variables were expressed with ($\bar{x}$ ± s). The comparison between numerical variables was performed with *t* test or ANOVA; Values were expressed as mean ± SD (Standard Deviation); *p* < 0.05 means statistically significant.

## Abbreviations

MsPGN: mesangial proliferative glomerulonephritis
Irb: irbesartan
ESRD: end-stage renal disease
PCNA: proliferating cell nuclear antigen
TLRs: toll-like receptors
SHL: low-dose of Shenhua Tablet
SHM: medium-dose of Shenhua Tablet
SHH: high-dose of Shenhua Tablet
